# Silicone stent placement using a rigid bronchoscope via a tracheostoma in a patient with postpneumonectomy syndrome

**DOI:** 10.1186/s44215-022-00015-0

**Published:** 2023-01-04

**Authors:** Kazuto Sugai, Naohiro Kobayashi, Yusuke Saeki, Shinji Kikuchi, Yukinobu Goto, Hideo Ichimura, Yukio Sato

**Affiliations:** Department of General Thoracic Surgery, University of Tsukuba Hospital, 2-1-1 Amakubo, Tsukuba, 305-8576 Ibaraki, Japan

**Keywords:** Postpneumonectomy syndrome, Stent placement, Rigid bronchoscopy, Tracheostoma

## Abstract

**Background:**

Silicone stents are options for airway stenosis or obstruction. Generally, silicone stent placement needs rigid bronchoscopy, but manipulation of a rigid bronchoscope has technical difficulties in some cases. The objective of this clinical case report is to highlight silicone stent placement with a rigid bronchoscope successfully achieved by changing the insertion from the mouth to the tracheostoma in a patient with postpneumonectomy syndrome.

**Case presentation:**

A 47-year-old man underwent right-side pneumonectomy 30 years previously, and he had an intubation tube inserted through a tracheostoma for 29 years to maintain the lumen for bronchial stenosis due to postpneumonectomy syndrome. A granuloma grew at the distal end of intubation tube, and he had labored breathing with stridor. Rigid bronchoscopy for silicone stent placement via the mouth failed because of airway curvature. We sequentially tried rigid bronchoscopy via the tracheostoma as an alternative, and we could easily insert the scope into the left secondary carina and place the silicone stent.

**Conclusion:**

Although strong airway curvature with tracheostomy is an uncommon situation, we succeeded silicone stent placement using a rigid bronchoscope via the tracheostoma. Insertion of a rigid bronchoscope via the tracheostoma instead of via the mouth could be an option.

## Background

Postpneumonectomy syndrome (PPS) is a rare complication of dynamic airway obstruction due to mediastinal shift, which sometimes leads to fatal respiratory failure. Although the treatment for PPS has not been established, stent placement is one method for lifesaving. Generally, silicone stent placement needs rigid bronchoscopy via the mouth, but manipulation of a rigid bronchoscope has technical difficulties and needs expertise. Furthermore, insertion of a rigid bronchoscope into the peripheral left main bronchus in patients with strong airway curvature is more difficult. Herein, we report successful silicone stent placement into the left main bronchus with a rigid bronchoscope via a tracheostoma in a patient with PPS.

## Case presentation

A 47-year-old man sustained a right bronchial laceration in a traffic accident and underwent right-side pneumonectomy at a former hospital 30 years previously. One year after the operation, he was transported to our hospital because of dyspnea. CT showed that the left main bronchus was stretched and pressed between the pulmonary artery and the T4-6 vertebral body, rendering the bronchus extremely stenotic, and PPS was diagnosed. Although an anterior resection of the thoracic vertebral body was performed to release the bronchus, the dyspnea did not improve. A tracheostomy was performed and an intubation tube (inner diameter, 7.0 mm; length, 20 cm) was placed from the tracheostoma to the left main bronchus to retain the airway lumen. The intubation tube was exchanged every half year to prevent obstruction. Thirty years after the pneumonectomy, the patient visited the hospital with dyspnea. Bronchoscopy showed a granuloma at the distal end of the intubation tube and that the left main bronchus was barely open. The granuloma was cauterized by use of neodymium-doped yttrium aluminum garnet laser and argon plasma coagulation, but it regrew. Although mitomycin was used and radiotherapy (30 Gy/15 Fr) was performed to shrink the granuloma and keep the bronchus open, the bronchial stenosis did not improve. The patient’s dyspnea continued to worsen, and he was transferred to our hospital for further treatment.

**Fig. 1:**
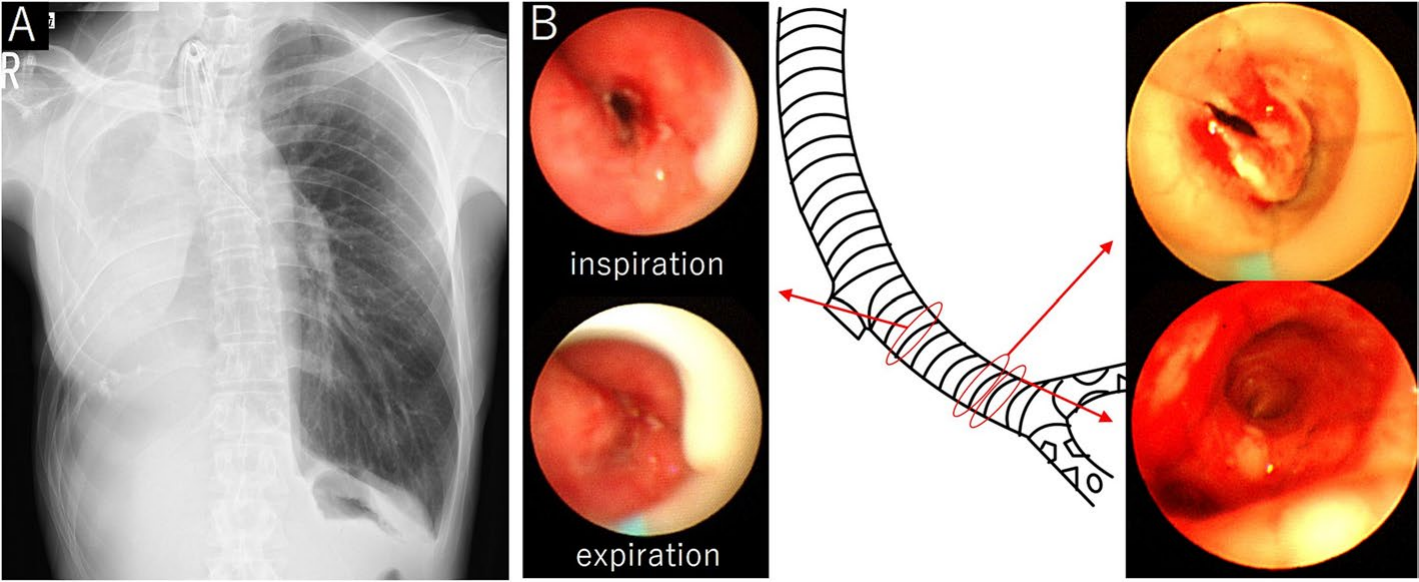
**A** Chest X-ray at the time of transfer to our hospital. The mediastinum was shifted to the right, and the airway from the upper trachea to the left main bronchus was steeply curved. **B** Bronchoscopic findings and schema of the trachea and the left bronchus. At the proximal side of the left main bronchus, the airway was barely patent during inspiration but was closed during expiration, which was caused by bronchomalacia. At the distal side of the left main bronchus, bronchial stenosis due to granuloma was revealed, but the secondary carina was intact

**Fig. 2:**
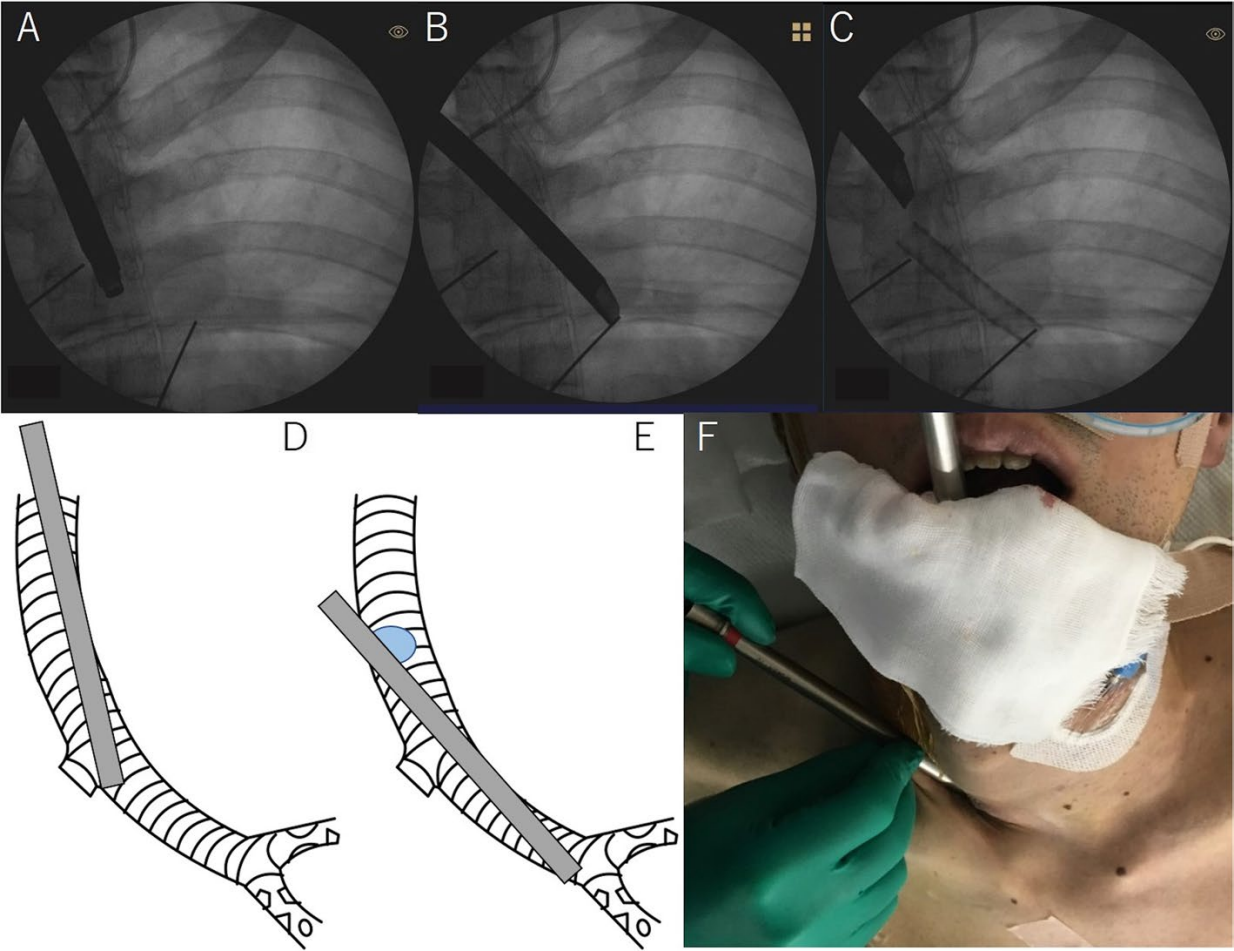
Fluoroscopic radiographs (**A**–**C**), schemas (**D**, **F**), and photograph (**F**) taken during the stent placement with a rigid bronchoscope. **A** and **D** show bronchoscopy via the mouth and **B**, **C**, and **E** bronchoscopy via the tracheostoma. **A** Rigid bronchoscope inserted via the mouth failed to reach to the distal left main bronchus. **B**, **C** Rigid bronchoscope inserted via the tracheostoma reached the secondary carina and led to successful stent placement. **D**–**F** There was a difference in the angle between the rigid bronchoscopy inserted via the mouth and that inserted via the tracheostoma

Although his oxygen saturation remained above 94% without oxygen supply, the patient had severe labored breathing with stridor, and frequent sputum suction was required. He was deemed to be at high risk of asphyxiation, so we introduced extracorporeal membrane oxygenation (ECMO). Drainage and return cannulas were inserted from the right internal jugular vein and the right femoral vein, respectively. After stabilization of blood oxygenation, the intubation tube was removed and bronchoscopy was performed, which revealed that the left bronchus was narrowed 2 cm distal from the carina over about 4 cm because of bronchomalacia, and a granuloma was detected 5 mm proximally to the secondary carina (Fig. 1). After cauterizing the granuloma with the hot biopsy forceps technique to dilate the bronchus, we tried placement of a silicone stent with rigid bronchoscopy via the mouth, but the scope could not reach the distal end of the left main bronchus owing to tracheobronchial curvature. Therefore, we tried again via the tracheostoma, and we could easily insert the scope into the left secondary carina and place the silicone stent (straight-shaped; inner diameter, 10 mm; length, 6 cm; Dumon Tube BD, Novatech, Aubagne, France) (Fig. 2). The ECMO was removed 3 days after the stent placement because frequent bronchoscopic sputum aspiration was required. Although nebulizers and expectorants for sputum were needed, the patient lived without dyspnea for 2 years after the stent placement.

## Discussion

In the current case, we performed rigid bronchoscopy via a tracheostoma and easily placed a silicone stent in the left main bronchus of a patient with PPS. A rigid bronchoscope is generally inserted into the trachea and bronchus via the mouth. However, the procedure is sometimes difficult because of airway curvature, neck flexion restriction, and limited mouth opening. In such cases, several techniques of silicone stent placement via a tracheostoma without rigid bronchoscopy have been reported. Nomori et al. reported a method of filling a silicone stent in an endobronchial tube and introducing it into the stenosis site by use of a pusher [1]. Hayashi et al. reported a method of placing a modified silicone stent through a tracheostoma by use of a forceps [2]. These methods do not require a rigid bronchoscope, which makes stent transport to the peripheral bronchi easier but can be difficult to adjust the stent position. In our case, the rigid bronchoscopy could not be inserted into the peripheral left main bronchus via the mouth because of strong airway curvature owing to the upper part of the trachea being shifted to the right side. Therefore, the rigid bronchoscope was inserted through the tracheostoma, which made the airway to the left main bronchus straighten easily. The successful points in our case were as follows: (a) the diameter of tracheostoma was large enough to insert a rigid bronchoscope, (b) the angle between the tracheostomy and the tracheal inlet was gradual, and (c) the shape of the airway from the tracheostoma to the left main bronchus was a gentle curve. This technique might be helpful in patients in whom the procedure of rigid bronchoscopy via the mouth is difficult owing to airway deformity, obesity, limited mouth opening, or limitation of head retroflexion. However, a new tracheostomy to insert a rigid bronchoscope should be carefully considered on a case-by case basis. In addition, potential complications such as hemorrhage and injury to the surrounding tissue should be noted.

PPS is a rare complication after pneumonectomy (occurring in 0.16–2% of cases) [3, 4] and is caused by a severe mediastinal shift. Most patients with PPS complain of progressive dyspnea on exertion and shortness of breath with stridor, eventually followed by respiratory failure and tracheomalacia due to the long-term pressure [3, 4]. Treatment for PPS could be divided into 2 strategies: mediastinum repositioning or lumen retention. In this case, anterior resection of a vertebral body and intubation tube placement were selected for lumen retention at the former hospital. Eventually, however, long-term placement of the tube led to formation of a granuloma and airway obstruction, and the patient was transferred to our hospital. Considering the long-term course and bronchomalacia, we chose stent placement instead of repositioning and fixation of the mediastinum by implantation of a thoracic prosthesis.

In conclusion, rigid bronchoscopy via a tracheostoma led to successful silicone stent placement in the current case with PPS. The technique of rigid bronchoscopy via a tracheostoma can be a useful option when rigid bronchoscopy via the mouth is difficult or fails.

## Data Availability

Not applicable.

## Abbreviations

PPS: Postpneumonectomy syndrome
CT: Computed tomography
ECMO: Extracorporeal membrane oxygenation
